# Supplementary material for: The interactome of intact mitochondria by cross-linking mass spectrometry provides evidence for coexisting respiratory supercomplexes
Source: Mol Cell Proteomics. 2017 Dec 8;17(2):216–32. doi: 10.1074/mcp.RA117.000470 (PMC5795388; doi:10.1074/mcp.RA117.000470)
Supplement: Supplemental Data [file supp_RA117.000470_133922_0_supp_23978_fzffwf.zip › spectra_annotation/mito_DR_spectra_annotation/482-1-18-1-20-1.pdf]

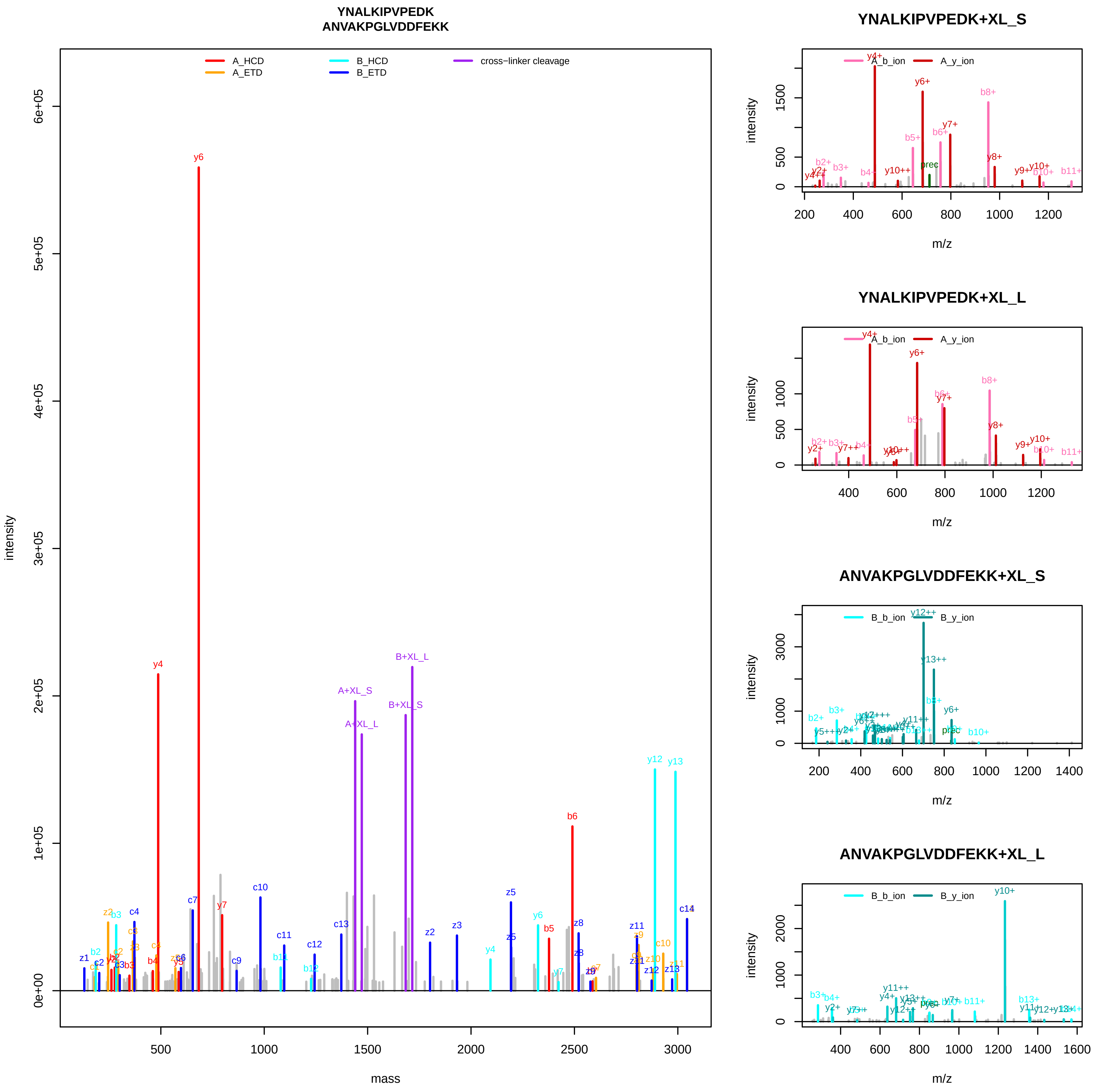
YNALKIPVPEDK
ANVAKPGLVDDFEKK
A_HCD
A_ETD
B_HCD
B_ETD
cross−linker cleavage
intensity
mass
0e+00
1e+05
2e+05
3e+05
4e+05
5e+05
6e+05
500
1000
1500
2000
2500
3000
y6
y4
A+XL_S
A+XL_L
B+XL_L
B+XL_S
y12
y13
b6
c10
c14
z5
y7
c7
c4
c11
c12
c13
z2
z3
z8
z11
YNALKIPVPEDK+XL_S
YNALKIPVPEDK+XL_L
ANVAKPGLVDDFEKK+XL_S
ANVAKPGLVDDFEKK+XL_L
A_b_ion
A_y_ion
B_b_ion
B_y_ion
m/z
y4+
y6+
b8+
y7+
b6+
b5+
y8+
prec
y12++
y13++
y6+
y10+
0
500
1000
1500
2000
3000
200
400
600
800
1000
1200
1400
1600
